# Extraction and Isolation of Bioactive Compounds From *Malpighia glabra* L. Leaves: In Vivo Investigation of the Leaf Extract Against Oxidative Stress and Inflammation Triggered by γ‐Radiation

**DOI:** 10.1002/cbdv.71636

**Published:** 2026-09-08

**Authors:** Amany M. Fekry, Walaa A. El‐Sabbagh, Marwa S. Abu Bakr, Abd El‐Salam I. Mohammed, Mona A. El‐Ghazaly

**Affiliations:** ^1^ Drug Radiation Research Department National Center for Radiation Research and Technology (NCRRT) Egyptian Atomic Energy Authority (EAEA) Cairo Egypt; ^2^ Department of Pharmacognosy, Faculty of Pharmacy (Girls) Al Azhar University Cairo Egypt; ^3^ Department of Pharmacognosy, Faculty of Pharmacy (Boys) Al Azhar University Cairo Egypt

**Keywords:** anti‐inflammatory, antioxidant, *Malphigia glabra* L., γ‐radiation

## Abstract

*Malpighia glabra* L. (Acerola), belonging to the family Malpighiaceae, is highly rich in phenolics, flavonoids and ascorbic acid content, which are collectively recognized for their potent antioxidant and anti‐inflammatory activities. Since ionizing radiation harm is primarily driven by excessive reactive oxygen species generation and persistent inflammation, this study aimed to explore the radioprotective potential of *Malpighia glabra* leaf extract (MLE) and identify its bioactive constituents. β‐isoarborinol, afzelechin, engeletin, and flavonoid glycosides had been isolated from the ethyl acetate fraction, and their structures were assigned based on NMR/MS data. Histopathological examination revealed that MLE treatment attenuated radiation‐induced histopathological alterations in multiple organs of irradiated rats. The anti‐inflammatory activity of MLE was elucidated by suppression of the intestinal NF‐κB/TNF‐α signaling cascade, along with systemic suppression of serum TNF‐α (>70%) and CRP (>25%). MLE restored redox homeostasis by normalizing GSH, TBARs and TAC levels, ultimately preserving hepatic and renal functions. Otherwise, MLE exhibited modest protective effects on haematological parameters, accompanied by dose‐dependent preservation of body weight. These findings identify MLE as a promising natural radioprotective agent and provide a mechanistic basis for its further development as an adjunct strategy to reduce radiation‐induced systemic and tissue alterations in a dose‐dependent manner.

## Graphical abstract

*Malpighia glabra* L. leaves extract, enriched with flavonoids and β‐isoarborinol, exhibits antioxidant and anti‐inflammatory activities that moderate γ‐radiation‐induced systemic and tissue harm. The extract preserves intestinal, hepatic, renal, splenic, and systemic integrity by reducing oxidative stress and inflammation, supporting its potential as a natural radioprotective extract.

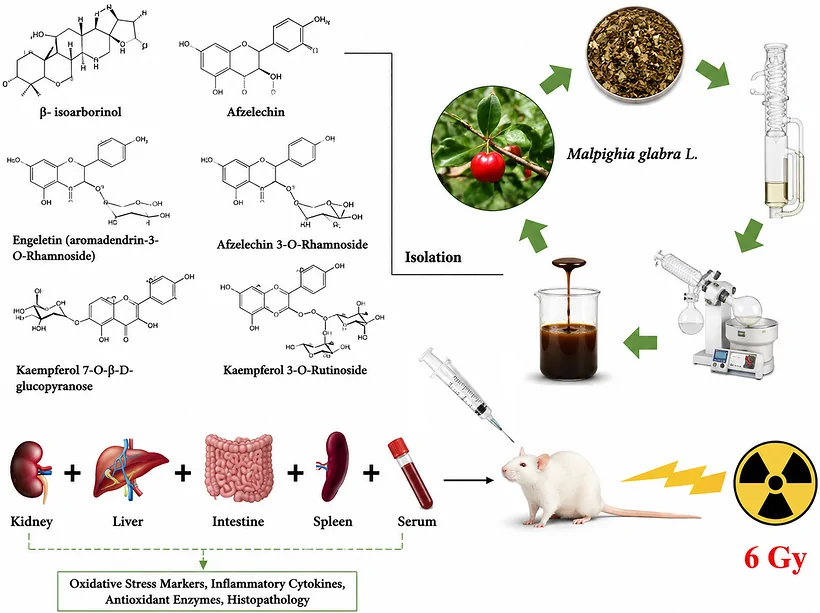

**Abbreviations**
ALT: alanine amino transferase enzyme
AST: aspartate amino transferase enzyme
Cr: creatinine
CRP: C‐reactive protein
Gy: gray
GSH: glutathione
TAC: total antioxidant capacity
TBARs: thiobarbituric reactive substances
TNF‐α: tumor necrosis factor alpha

## 1 Introduction

Employing of γ‐radiation in a cancer management technique carries many negative effects that could impair patients' quality of life. Numerous studies have challenged the ability of medicinal plants to protect normal tissue from the oxidative stress generated by γ‐radiation and its further complications [1]. The main pathological pathway of γ‐radiation on normal cells was attributed mainly to the production of reactive oxygen species (ROS) that interact with cellular macromolecules such as proteins, nucleic acids, and lipids, followed by the surging of inflammatory mediators as tumor necrosis factor and interleukins (TNF‐α, IL‐1β, IL‐6), and maintenance of inflammatory transcriptional factors such as nuclear factor kappa (NF‐kB), which exerted mitochondrial dysfunction and disrupted cell proliferation and differentiation, and finally commanded to cell apoptosis [2, 3].

The adverse effects of γ‐radiation exposure extended to many organs, but the first adverse action has been observed in hematopoietic system and gastrointestinal tract as it caused the suppression of bone marrow progenitor cells and destruction of intestinal crypt cells [4]. The acute radiation phase in the gastrointestinal tract emerged promptly within a few days post‐radiation exposure, significantly impacted by the extent of inflammation and dysbiosis, and clinically presented as acute enteritis, abdominal discomfort, and diarrhea [5, 6]. Moreover, hepato‐ and nephrotoxicity are additional adverse effects of ROS generation following γ‐radiation exposure, which may be mitigated by the consumption of powerful antioxidants [7].

Natural compounds have been widely investigated as radioprotective agents due to their ability to counteract radiation‐induced oxidative stress, inflammation, and DNA damage. Plant‐derived phytochemicals such as polyphenols, flavonoids, alkaloids, polysaccharides, and vitamins exert radioprotective effects primarily through free‐radical scavenging, enhancement of endogenous antioxidant defenses, modulation of inflammatory signaling, and promotion of DNA repair mechanisms. Polyphenolic compounds, including flavonoids such as quercetin and its glycosides, have been shown to reduce radiation‐induced cellular damage and enhance genomic stability in cell and animal models by attenuating oxidative stress and inflammatory responses. Natural antioxidants like vitamins C, E, and selenium also demonstrate radioprotective activity by scavenging reactive oxygen species and protecting chromosomal integrity [8, 9, 10, 11, 12, 13]. Collectively, these findings highlight the potential of natural products as low‐toxicity radioprotectors that may complement conventional radiotherapy and mitigate normal tissue damage.

*Malpighia glabra* L., belongs to Malpighiaceae family, is one of the richest plant sources of ascorbic acid and is also known as ‘acerola’. It is cultivated in tropical and subtropical regions, and the flowering period lasts from April to November every year. The fruit is used commercially as a dietary supplement to enhance the ascorbic acid content of other fruit juices. Acerola juice possesses the equivalent vitamin C content of 14 liters of orange juice, with ascorbic acid constituting around 3.5% of the weight of fresh fruit; furthermore, the vitamin C derived from acerola is more effectively absorbed by human tissue compared to synthetic ascorbic acid. Due to its abundance in antioxidants, minerals, and vitamins, Acerola has long been utilized as a popular antifungal, antiemetic, and immune‐enhancer plant. Additionally, cherry fruit is rich in carotenoids, anthocyanins, and vitamins [A, B1, and B2]. Previous studies on acerola fruit showed its richness of phenolic, flavonoid, anthocyanin, carotenoids in addition to ascorbic acid. These studies demonstrated acerola extract's efficacy as an anti‐mutagenic, anti‐inflammatory and radioprotective agen  [14, 15].

Although the fruits of *Malpighia glabra* L. have been extensively investigated for their antioxidant and radioprotective properties, the phytochemical composition and biological activities of its leaves remain largely unexplored. In particular, no previous study has comprehensively isolated and structurally characterized the major bioactive constituents of *Malpighia glabra* L. leaves while simultaneously evaluating their radioprotective efficacy and underlying molecular mechanisms in vivo, as leaves constitute a sustainable source of phytochemicals that may possess distinct biological activities from the fruits. However, few works were carried on the leaf part of *Malpighia glabra* L. and explored the powerful free radical scavenging activity of the leaf part extract and its potential hepatoprotective effect [16, 17]. In addition to high ascorbic acid content, *Malpighia glabra* L. leaves are also abundant with biologically active flavonoids such as quercetin, kaempferol and apigenin, in addition to coumarins [15], which are more chemically stable than ascorbic acid and exhibit potent antioxidant, anti‐inflammatory, and signaling‐modulatory properties [8, 13, 18].

Therefore, this study was designed to (i) isolate and characterize the major bioactive constituents of *Malpighia glabra* L. leaves, (ii) investigate the radioprotective efficacy of the whole aqueous leaves extract in γ‐irradiated rats, and (iii) elucidate the underlying mechanisms by evaluating oxidative stress, systemic and intestinal inflammatory responses, histopathological alterations, and modulation of the NF‐κB/TNF‐α signaling pathway.

## 2 Material and Methods

### 2.1 Sample Preparation

Fresh leaves (1.5 kg) were collected from the *Malphigia* *glabra L*. tree cultivated in Orman Garden, Giza, Egypt, and were authenticated by Mrs Teresa Labib, Head of the “Taxonomists at Orman Botanic Garden”. A voucher specimen was prepared and deposited in the Herbarium of the Faculty of Pharmacy (Girls), Al‐Azhar University, Egypt, under voucher specimen code MG017, where it is available for future reference and verification. Leaves were powdered using a mill to fine powder, then extracted by 99% MeOH successive times to give and concentrated under vacuum by rotavapor device to give 300 g crude extract. The crude extract was defatted by petroleum ether solvent successively to get rid of undesired fatty substances; the defatted extract was dissolved in distilled water, and precipitation processes were done by the addition of ethanol solvent to precipitate sugar/polysaccharides to purify the extract [19]. The extract after precipitation was dissolved in 500 mL of distilled water and fractionated by petroleum ether, ethyl acetate and *n*‐butanol solvents 3 successive times, which are summarized as follows:

The ethyl acetate fraction was concentrated to give 9 g of extract and injected into a silica gel (200 g) column and eluted by methylene chloride/methanol (100:0 to 50:50) to give five collective fractions. Finally, the collective fractions were subjected to Sephadex LH‐20 column chromatography that was MeOH:MeCl_2_ (100:0) to (95:5) to give 6 pure compounds.

Fraction A was subjected to CC using Sephadex LH‐20 and eluted with MeOH:MeCl_2_ (90:10) to (60:40), and TLC systems were used to collect (4‐39) to give pure compound 1, while fraction B was subjected to CC using Sephadex LH‐20 and eluted with 100% MeOH, and TLC systems were used to collect (14‐18), which were injected again into CC Sephadex LH‐20 to give (17‐20) pure compound 2. Fraction C was subjected to CC using Sephadex LH‐20 and eluted with 100% MeOH, and TLC systems were used to collect 25‐28, which were subjected again to CC Sephadex LH‐20 to give (15‐28) pure compound 3. Fraction D was subjected to CC using Sephadex LH‐20 and eluted with 97.5:2.5 MeOH:MeCl_2_, and TLC systems were used to collect (32‐39) and (48‐52) which were subjected again to CC Sephadex LH‐20 to give (6‐15) pure compound 4 and (24‐30) pure compound 5, and fraction E was subjected to CC using Sephadex LH‐20 and eluted with 95:5 MeOH:MeCl_2_, and TLC systems were used to collect (22‐30), which were subjected again to CC Sephadex LH‐20 to give (25‐35) pure compound 6.

Si gel (Si gel 60, Sigma, Aldrich, Egypt) and Sephadex LH‐20 (Sigma, Aldrich, Egypt) were used for open column chromatography. Solid phase extraction was performed on SPE‐C18 cartridges (Strata columns). TLC was carried out on precoated silica gel 60 F254 (Sigma, Aldrich, Egypt) plates, then visualized after spraying with 1% vanillin‐H _2_SO_4_, followed by heating at 1000°C for 5 min.

### 2.2 Mass and NMR Spectrum

**FIGURE 1:**
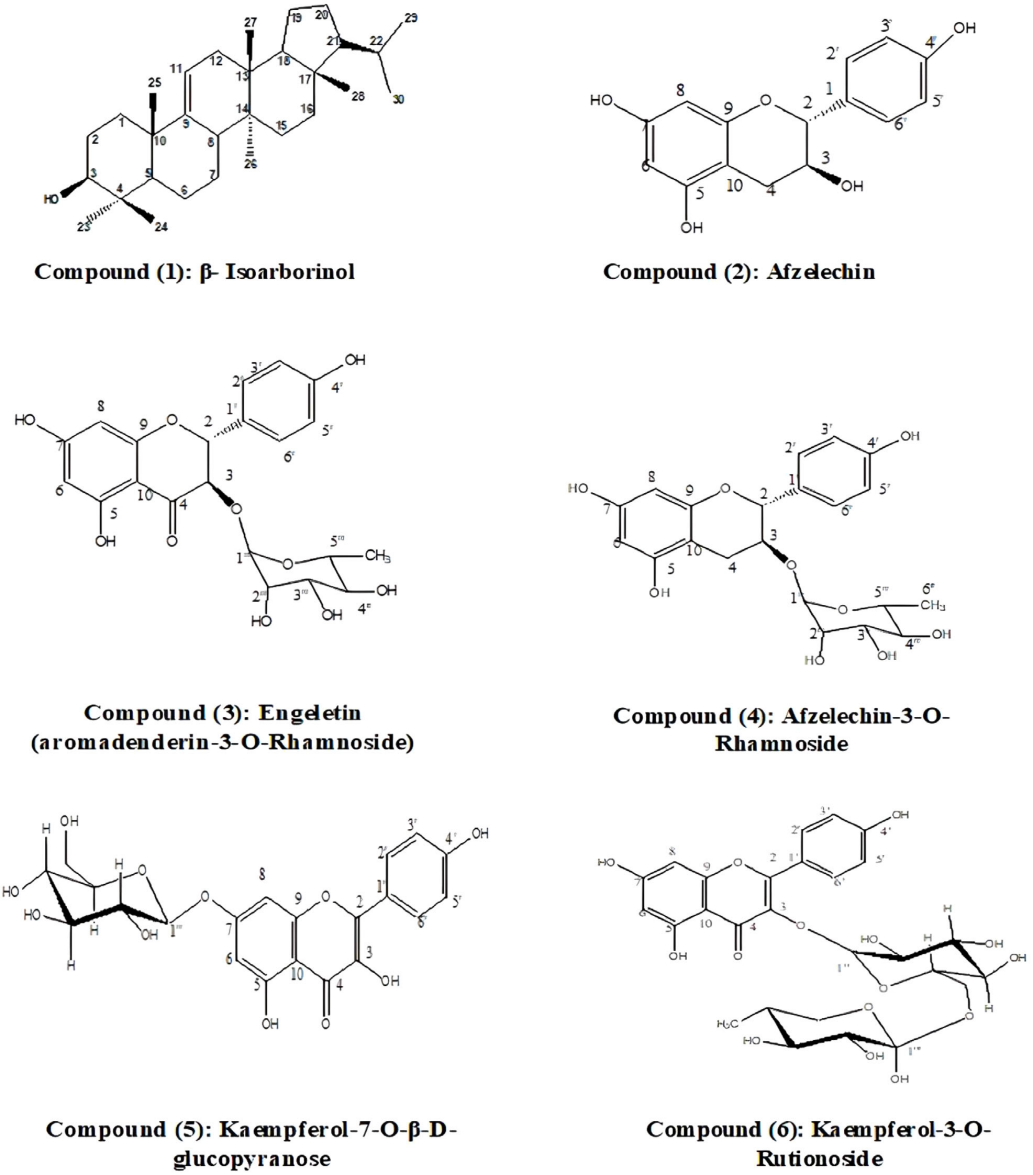
Chemical structures of MLE isolated compounds. Structures assignment based on NMR spectral data and mass spectrometer analysis.

**FIGURE 2:**
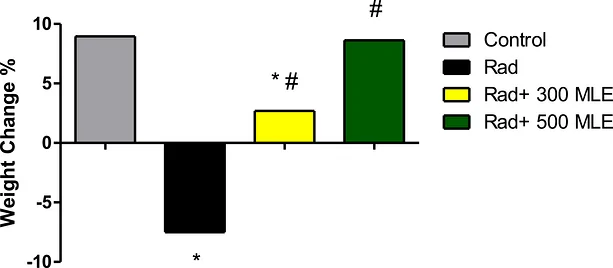
Effect of MLE (300 and 500 mg/kg, p.o./5 days) on percentage body weight changes of 6 Gy irradiated rats (*n* = 6). Data were analyzed by one‐way ANOVA followed by a Tukey post‐hoc test. * Significant from the control group at *p* < 0.05. # Significant from the Rad group at *p* < 0.05.

**FIGURE 3:**
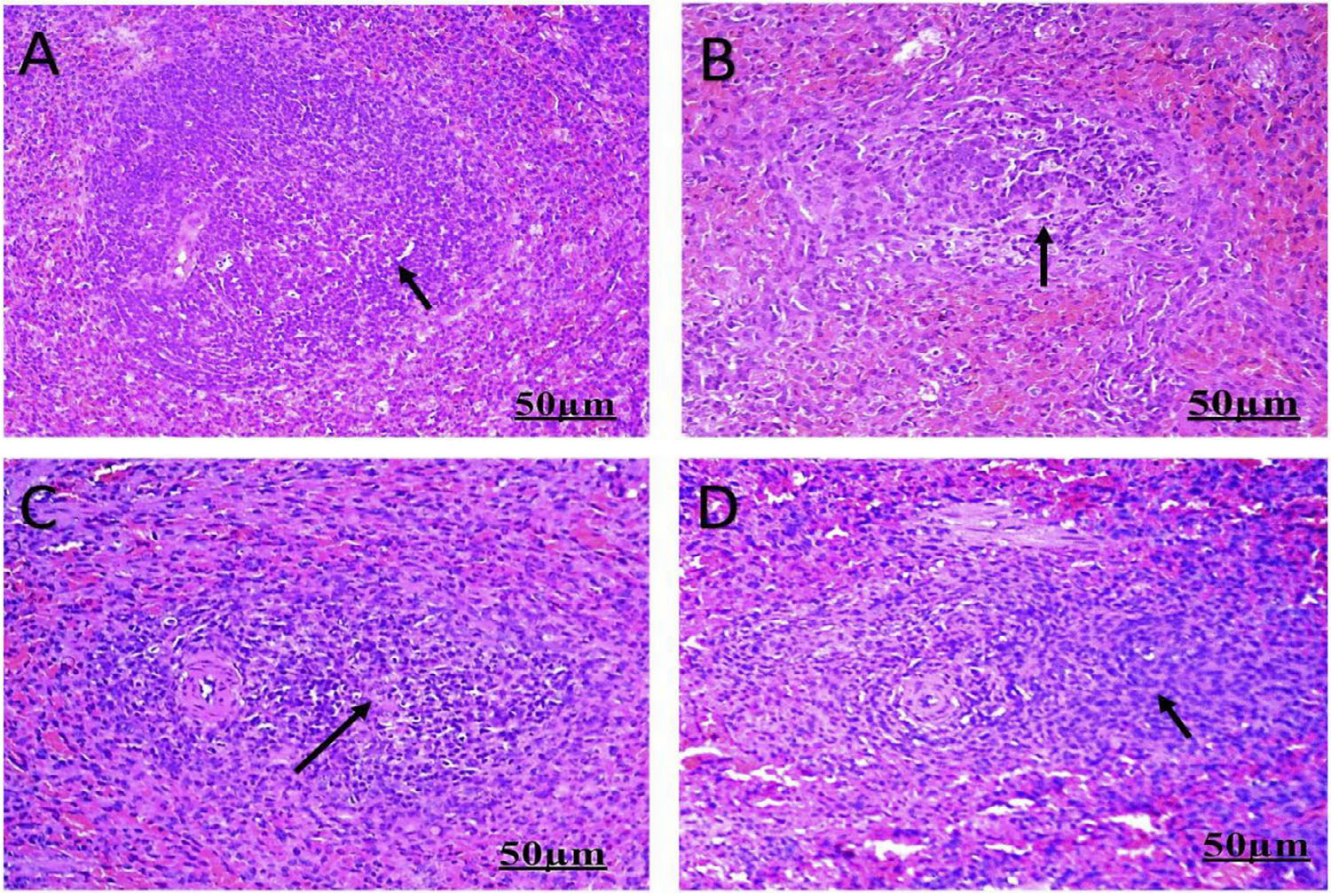
Photomicrograph of splenic tissue section showing (A) normal splenic architecture with its two major components, white pulp and red pulp, in the control x000A0;group; (B) depletion of white pulp and indistinct marginal zones in the Rad group; (C) moderate to severe depletion of white pulp follicle cells and congestion of splenic sinuses in the Rad ± 300 MLE group. (D) Few numbers of congested splenic sinuses with prominent periarteriolar lymphocytic sheaths in the Rad ± 500 MLE group (H&EX200).

**FIGURE 4:**
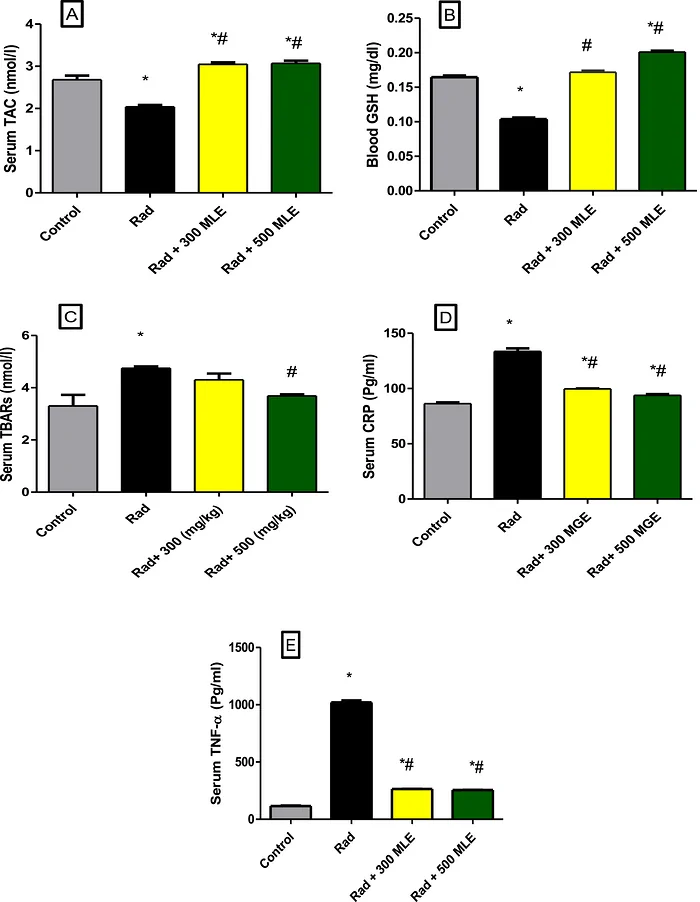
Effect of MLE (300, 500 mg/kg, p.o) on serum [A] TAC (nmol/l), [B] GSH (mg/dl), [C] TBARs (nmol/l), [D] CRP (pg/ml), and [E] TNF‐α (pg/ml) of 6 Gy irradited rats (*n* = 6). Data were analyzed by one way ANOVA followed by Tukey post‐hoc test. * Significant from control group at *p* < 0.05. # Significant from Rad group at *p* < 0.05.

**FIGURE 5:**
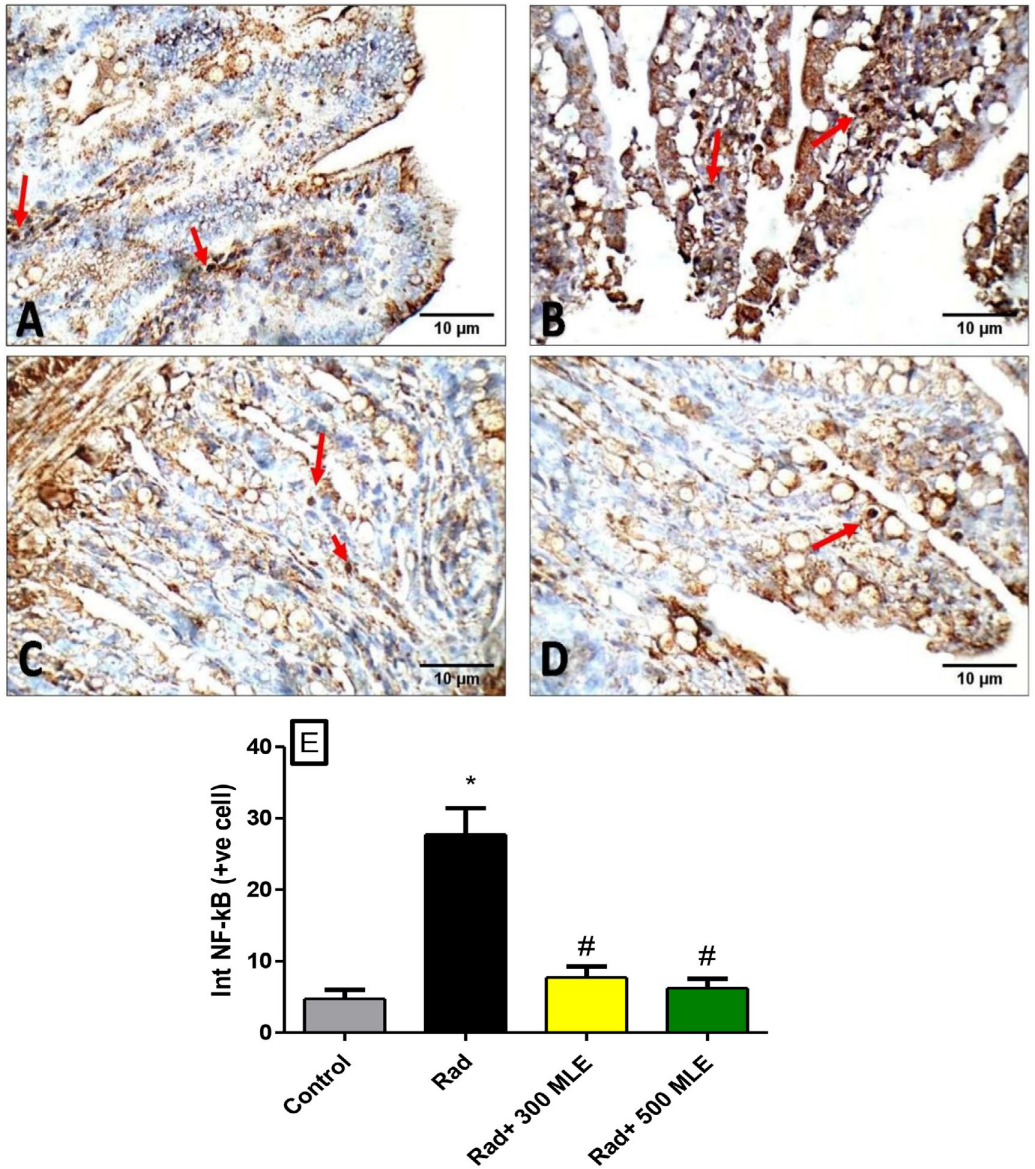
Immunohistochemical examination of NF‐κB protein expression in rats' intestinal tissue after 6 Gy irradiation. [A] Normal group showing a limited number of NF‐κB positive cells; [B] Rad group showing high intense expression of NF‐κB positive cells; [C] Rad + 300 MLE and [D] Rad + 500 MLE groups showing significant lower expression of NF‐κB positive cells; [E] NF‐κB expression in lung‐examined areas. Data were analyzed by one‐way ANOVA followed by a Tukey post‐hoc test. * Significant from the control group at *p* < 0.05. # Significant from the Rad group at *p* < 0.05.

**FIGURE 6:**
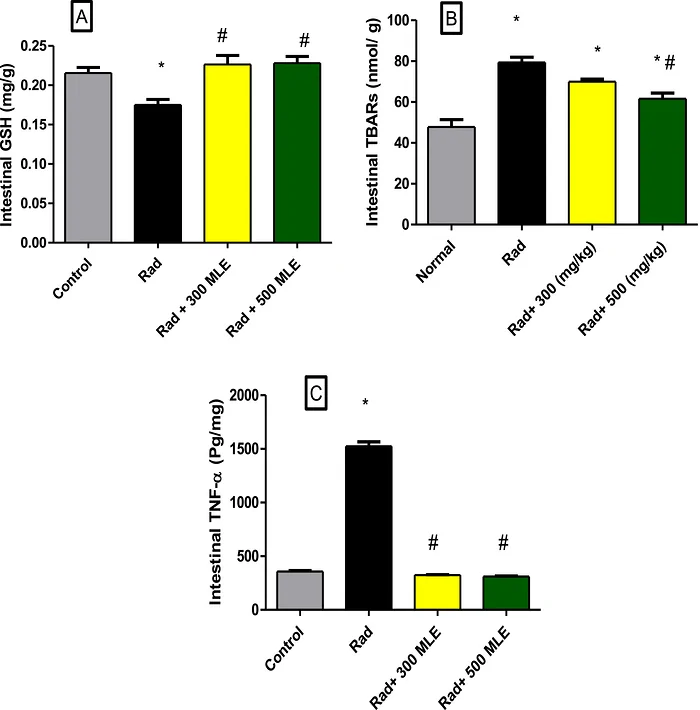
Effect of MLE (300 and 500 mg/kg, p.o.) on intestinal [A] GSH (mg/g), [B] TBARs (nmol/g), and [C] TNF‐α (pg/mg) of 6 Gy irradiated rats (*n* = 6). Data were analyzed by one‐way ANOVA followed by a Tukey post‐hoc test. * Significant from the control group at *p* < 0.05. # Significant from the Rad group at *p* < 0.05.

**FIGURE 7:**
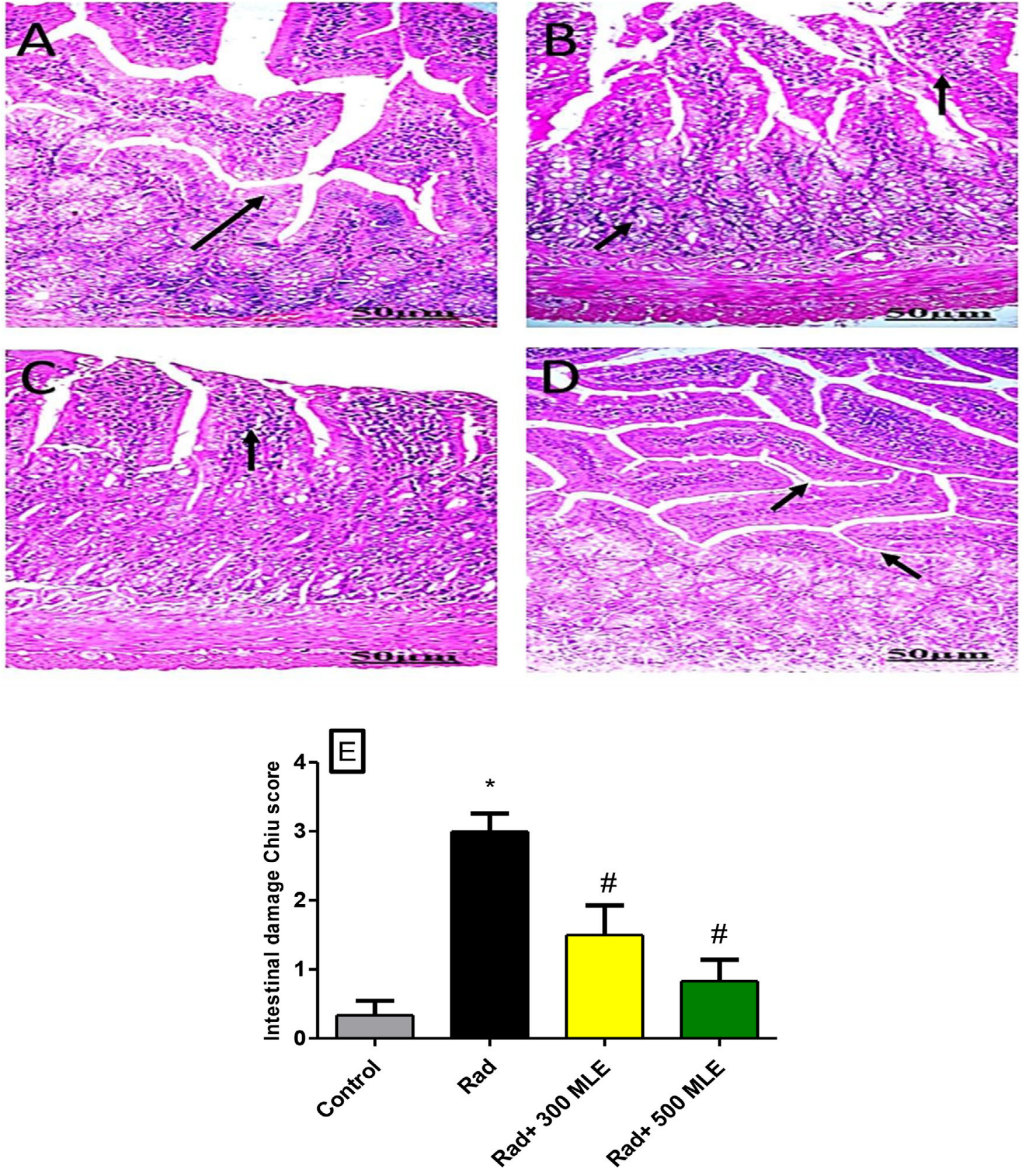
Photomicrograph of intestinal mucosa tissue section showing (A) normal histological structure of intestinal mucosa in control group, (B) shortening and atrophy of some villi with marked sub‐epithelial edema and sloughing of villi epithelial lining in Rad group, (C) shortening and atrophy of intestinal villi with leukocytic infiltration, moderate sloughing of the villus epithelial lining and a moderate loss of goblet cells in the Rad ± 300 MLE group, (D) mild sub‐epithelial edema and intact villus epithelial linings with minimal loss of goblet cells in the Rad ± 500 MLE group (H&E X200), and (E) intestinal damage Chiu scoring (*n* = 6). Data were analyzed by one‐way ANOVA followed by a Tukey post‐hoc test. * Significant from the control group at *p* < 0.05. # Significant from the Rad group at *p* < 0.05.

ESI‐MS was measured on a TQD triple quadruple instrument (Waters Corporation, Milford, instrument), Figure 7S. NMR spectra (Figures 1S‐6S) were recorded on a Varian Mercury 400 or JEOL 500 spectrophotometers at 400 for H^1^ and 100 MHz for C^13^, in DMSO‐d6 solution, and chemical shifts were expressed in δ (ppm) with reference to TMS and coupling constant (J) in Hertz.

### 2.3 Phytochemistry Investigations

Chromatographic investigations of MLE yielded six pure compounds, the first time to be isolated from *Malpighia glabra* L. leaves and the family Malpighiaceae: compound 1 (ß‐isoarborinol), compound 2 (afzelechin), compound 3 (engeletin), compound 4 (afzelechin 3‐O‐rhamnoside), compound 5 (Kaempferol 7‐O‐β‐D‐glucopyranose) and compound 6 (Kaempferol 3‐O‐Rutinoside) (Figure 1).

#### 2.3.1 Compound 1 (β‐isoarborinol)

It was isolated as white crystals with a melting point of 295‐298°. The ESI‐MS showed a molecular ion peak at *m/z* 426 for [M]⁺.

The ^1^H NMR spectrum (Table S1) exhibited a triterpene profile, characterized by six singlets at low frequency: δH 0.78, 0.78, 0.85, 0.85, 0.92, and 1.02 were assigned to six methyl groups of C‐28, C‐27, C‐26, C‐24, C‐23, and C‐25, respectively, and two multiplet methyls at δH 0.83 and 0.89 corresponding to the two methyl groups of the isopropyl fragment. Furthermore, one olefinic proton (δH 5.11 (1H, brs, H‐11), and one oxymethinic proton δH 3.50 (1H, dd, H‐3β) with large coupling constant (*J* = 9.5 Hz) between H‐3 and H‐2, indicates their axial disposition, thereby indicating that the hydroxyl group at the C‐3 is equatorially oriented [20].

The presence of 30 signals on ^13^C NMR including eight methyl's, nine methylene's, seven methine (including one *sp2* at δC 114.57 and one *sp3* oxygenated at δC 78.34) and six quaternary carbon atoms.

The HMBC spectrum showed correlations between the signal at δ 0.89 (H‐29) and the signals at δ 59.94, 31.20, and 22.69 corresponding to (C‐21, 22, 30), and between the signal at δH 0.83 (H‐30) and the signals at δ 59.94, 31.20, and 23.42 corresponding to (C‐21, 22, 29); permitting assignment of the isopropyl moiety. The signal at δ 5.11 was assigned to the olefinic proton H‐11, owing to correlation with the signal at δ 41.43 (C‐8) and the signal at δ 3.50 was assigned to the oxymethinic proton H‐3, owing to correlation with the signal at δ 29.17 and 16.84 corresponding to (C‐23, 24).

From the abovementioned data and by comparison of spectroscopic data with previously reported spectra [21] compound 1 was identified as the pentacyclic triterpenoid β‐isoarborinol, a hopane triterpene which corresponded to the molecular formula of C_30_H_50_O.

#### 2.3.2 Compound 2 (Afzelechin)

It was obtained as bright yellow needles with 252–254°C melting point. It showed dark‐blue color with ferric chloride reagent and orange red color with vanillin sulfuric acid reagent. Its positive ion mode ESI‐MS showed a molecular ion peak at m/z 274 [M]^+^.

The ^1^H NMR spectrum (Table S2) provided twelve signals ranging from aliphatic to aromatic, characteristic for flavan‐3‐ol. One set of meta‐coupled protons at δ 5.68 (1H, br s, H‐6) and 5.89 (1H, br s, H‐8), was assigned to the protons of the A ring. The disubstituted aromatic B‐ring appeared as AA`BB` spin system at δ 6.73 (H‐3`, H‐5`) and δ 7.14 (H‐2`, H‐6`), each 2H, d, *J* = 8 Hz. The aliphatic region of the ^1^H NMR spectrum (4.5 to 2.4 ppm) showed an ABMX spin system reminiscent of the heterocyclic C‐ring protons; the signal at 4.52 ppm (1H, d, *j* = 8) was assigned to H‐2 due to the close correlation of the chemical shift to that of the literature value for a proton between an ether and phenyl group, the signal at 3.88 ppm was assigned to H‐3 (1H, m) and signals at δ 2.70(1H, dd, *J* = 16.0, 5.2 Hz), δ 2.35 (1H, dd, J = 16.0, 5.2 Hz), were assigned to H‐4α and H‐4β, respectively. The remaining four signals at δ 4.87, 8.94, 9.19, and 9.37 were assigned to the four hydroxyl groups. The higher field shifted signals at δ 3.86 (H‐3) and δ 4.52(H‐2) suggested the presence of a flavan‐3‐ol moiety with 2,3‐trans type stereochemistry. The small coupling constant between H‐2 and H‐3 and the ^13^C NMR chemical shifts for C‐2 (δ 80.98) and C3 (δ 66.28) were in agreement with those of afzelechin structure.

The ^13^C NMR spectrum offers confirmation of only 13 peaks for the 15 carbons, as expected. The upfield signals at δ 80.98(C‐2), 66.28 (C‐3) and 28.27 (C‐4) ppm are associated with the heterocyclic C‐ring aliphatic carbons. Signals for the aromatic A‐ring and B‐ring of a flavan‐3‐ol were observed in the downfield region (94‐157 ppm), δ 156.14(C‐5), 95.16(C‐6), 156.47(C‐7), 93.85(C‐8), 155.35(C‐9), 99.13(C‐10), 129.94(C‐1’), 128.56 (C‐2’/6’), 114.78 (C‐3’/5’) and 156.93 for (C‐4’).

The resonances for C– 3`/C–5` and C–2`/C–6` were observed by the same chemical shifts, confirming the p‐substitution of B‐ring. These data concurred with afzelechin molecular formula of C_15_H_14_O_5_. On the basis of the above data compound **2** was identified as afzelechin and its spectral data were in good agreement with those reported previously [22].

#### 2.3.3 Compound 3 (Engeletin)

It was isolated as white powder. The ESI‐MS showed a molecular ion peak at (m/z): 435.1 [M + H]^+^, it's also known as (2R,3R)‐ dihydro‐kaempferol 3‐R‐L‐rhamnopyranoside.

The ^1^H and ^13^C NMR of data is in accordance to a flavanone glycoside and characterized as 5,7,4’‐trihydroxy flavanone‐3‐O‐α‐L‐rhamnopyranoside or engeletin [23].

In the ^1^H NMR (400 MHz, DMSO‐d6) spectrum (Table S2), a pair of doubles (each for one proton) at 4.74 and 5.28 with *J* = 10.4 Hz suggested it to be a dihydroflavonol or its glycoside. Apart from these two proton signals, the ^1^H NMR spectrum clearly showed two doublets of four protons at 6.79 and 7.33 with *J* = 8.4 Hz, typical of the A_2_B_2_ pattern for the B‐ring [23] a singlet at 1.05 for methyl group, indicating the presence of a methyl group of rhamnose. Singlet peaks at 5.90 and 5.88 were assigned for C‐6 and C‐8, respectively.

The ^13^C NMR spectra of other dihydro‐flavanols and kaempferol in the literature with consideration of solvent effects. The signals of C‐6 and C‐8 in aromadendrin were obtained at 96.23 and 95.24. From the previous data and literature [24], compound 3 was identified as Engeletin.

#### 2.3.4 Compound 4 (Afzelechin 3‐O‐Rhamnoside)

It was isolated as yellow color. The ESI‐MS showed a molecular ion peak at *m*/*z* {420}.^1^H and ^13^C NMR spectra readily determined examination of the 5,7,4’‐trihydroxy substitution pattern was clearly discernible and comparison with relevant data leaves no doubt that the Rhamnoside moiety is situated at position 3.

^1^H NMR data (Table S2) reveled signals at 5.15 ppm (1H, d, *j* = 6.8) was assigned to H‐2, the signal at 4.28 ppm was assigned to H‐3 (1H, brs) and signals at δ 3.22 (1H, m, d, 11.6), δ 3.0 (1H, m), were assigned to H‐4α and H‐4β, respectively. The remaining four signals at δ 5.25, 9.53, 9.79 and 9.92 (1 h, brs) were assigned to the four hydroxyl groups. The higher field shifted signals at δ 4.28 (H‐3) and δ 5.15 (H‐2) suggested the presence of a flavan‐3‐ol moiety with 2,3‐trans type stereochemistry.

Two Broad singlet signals at 6.23 and 6.41 are assigned to (H‐6) and (H‐8), respectively.

(H‐2’/6’) and (H‐3’/5’) appeared at (7.64, d, *j* = 7.6) (7.23, d, *j* = 7.2), respectively.

The typical anomeric proton resonance at 4.66, the methyl resonance at δ 1.51 and the characteristic chemical shift positions of the remaining carbons on the sugar moiety served to confirm the L‐rhamnose residue.

^13^C NMR chemical shifts for C‐2 (δ 79.44) and C3 (δ 73.58) were in agreement with those of afzelechin structure [25]. The stereochemistry of substituents on ring C is trans as shown by the signal from H‐2 appearing as a doublet (*J* = 6.8 Hz) at 5.15 and was fully characterized and serves to confirm the structure of the title compound as (+)—afzelechin and 3‐O‐r‐L‐rhamnopyranoside, signals appeared at 156.76 (C‐5), 96.02(C‐6), 157.18(C‐7), 94.63(C‐8) corresponding to ring A, ring B signals appeared at 129.79(C‐1’), 128.79 (C‐2’/6’), 115.61(C‐3’/5’) 157.57(C‐4’).

#### 2.3.5 Compound 5 (Kaempferol‐7‐O‐β‐D‐glucopyranoside)

It was isolated as light‐yellow powder. The ESI‐MS data showed the protonated ion peak [M+H]^+^ at m/z 449. The ^1^H‐NMR spectrum showed at δ H 6.42 (1H, s, H‐6) and δ H 6.80 (s, H‐8) corresponding to C6‐ and C8–protons of the flavanol A ring. Two doublets at δ H 6.94 (2H, d, 8.4 Hz, H3’/H5’) and δH 8.08 (2H, d, 8.4 Hz, H2’/H6’) corresponded to their Para substituted flavonoid B‐ring.

The anomer proton signal at δ H 5.07 (1H, d, *J* = 6.8 Hz, H1') suggested that Compound (**5**) was a flavonoid glycoside.

The ^13^C‐NMR confirmed the presence of 21 carbon atoms. Besides the 15 carbon signals of the flavonoid nucleus, 147.18 (C‐2), 136.15 (C‐), 176.28 (C‐4), 160.55 (C‐5), 99.03 (C‐6), 166.15 (C‐7), 92.03 (C‐8), 155.89 (C‐9), 105.05 (C‐10), signals appeared at 121.41 (C‐1'), 129.74(C‐2'/6'), 115.33(C‐3'/5'), 159.55(C‐4’) of ring B (Table S2).

The ^13^C‐NMR spectrum also exhibited six carbon resonances of a sugar moiety, which appeared at 100.02(C‐1'), 72.99(C‐2'), 76.30 (C‐3''), 69.59(C‐4''), 77.24 (C‐5''), and 60.59(C‐6''); the correlation between the anomeric proton δ H 5.08 (H1'') and the carbon signal δ C 136.15 (C3) revealed that C3 is free and the linkage with the aglycone moiety is on C7. Based on above deductions and comparing spectral data [26], compound **5** was determined as **kaempferol‐7‐O‐β‐D glucopyranoside,** which matched with the molecular formula of C_21_H_20_O_11._

#### 2.3.6 Compound 6 (Kaempferol‐3‐O‐rutinoside)

It was isolated as yellow amorphous powder, m.p 199°C–201°C, gave positive Molisch's test with TLC investigation yielded one yellow spot on TLC changed to intensive yellow color after spraying, with AlCl_3_. ESI‐MS showed a molecular ion peak at m/z 594 {M}^+^ [27].

The ^1^H‐NMR spectrum (Table S2) revealed a clear Flavanol‐type pattern of aromatic proton signals; two meta‐coupled protons of one proton each at δ 6.19 ppm (s, H‐6), and δ 6.40 ppm (s, H‐8). The 4`‐oxygenated B‐ring (comprising the protons at C‐3`, ‐5`, and C‐2`, ‐6`) appeared as AA`BB` system, which showed the four‐peak pattern of two doublets at δ 6.88 ppm (H‐3`, 5`) and δ 7.98 ppm (H‐2`,6`) (with *J* value = 8 & 8.4 Hz), respectively. These are typically ortho‐related protons, indicating only one substituent at C‐4` (para disubstituted phenyl group).

The ^1^H‐NMR spectrum of compound **6** also exhibited two anomeric proton signals at δ 5.3 ppm (1H, d, *J* = 6.4 Hz, H‐1`` of glucose) and δ 4.38 ppm (1H, s, H‐1```of rhamnose), which were consistent with the configurations δ for D‐glucose and δ for L‐rhamnose, respectively in addition, the appearance of the strong sharp doublet at δ 0.98 ppm (3H, d, *J* = 6 Hz, CH_3_ of rhamnose) confirmed the rhamnose unit. The chemical shift value of the anomeric proton of glucose (δ 5.3 ppm, *J* = 6.4 Hz, β‐form). On the other hand, the chemical shift value of the anomeric proton of rhamnose (δ 4.38 ppm) confirmed that the rhamnose unit must be linked to the glucose moiety [28].

The ^13^C‐NMR spectrum of compound **6** supported these observations having the twenty‐seven carbon signals, 12 were arising from sugar moieties, glucose, and rhamnose. The remaining resonances were indicative for kaempferol as aglycone. The resonances for C‐3`/C‐5` and C‐2`/C‐6` were observed by the same chemical shifts [115.10, (C‐3`, C‐5`) and 130.88 (C‐2`, C‐6`)], confirming the p‐substitution of ring B [29].

Additionally, ^13^C‐NMR spectrum of compound **6** showed the presence of a disaccharide unit, which indicated by the anomeric carbons at δ 101.38 ppm (C‐1`` of glucose), 100.78 ppm (C‐1``` of rhamnose) and the signal at 17.74 (CH_3_ of rhamnose). The up‐field shift of C‐3 of the aglycone (133.21 ppm) indicates that the diglycosidic sugar moiety is attached to the position C‐3 of the aglycone (O‐glycosylation effect). The downfield shift of C‐6`` indicate the attachment of the rhamnose to C‐6`` of glucose moiety (rhamnosylation effect) [30]. These data ensured that this compound is (kaempferol‐3‐O‐rutinoside).

### 2.4 Biological Evaluation

#### 2.4.1 Experimental Animals

Male Swiss albino mice were used exclusively for the preliminary toxicological study. Male Wistar rats were used for the preliminary anti‐inflammatory screening and the main γ‐radiation‐induced injury study. All experiment had been conducted according to ARRIVE standards and the National Centre for Radiation Research and Technology (NCRRT) Animal Care Committee, Egyptian Atomic Energy Authority, Cairo, Egypt, has authorized the research ethics of animal care (Permit no: 4PA/24).

#### 2.4.2 Toxicity Study

An acute toxicity study was carried out on a total of 36 adult male Swiss albino mice (25–30g), where each group (*n* = 6) received a single oral dose of MLE (100, 200, 500, 800, 1000 and 2000 mg/kg body wt.), then observed for up to 14 days for any behavioural or weight changes or any mortalities.

#### 2.4.3 Screening of Anti‐inflammatory and Analgesic Activity in Carrageenan Induced Paw Edema Model

A total of 42 male Wistar rats (*n* = 6) were used in this screening study; 0.1 mL of 1% carrageenan (Sigma, Aldrich) was injected in right hind paw after 1 h from oral administration of MLE (150, 300, 500 or 1000 mg/kg), in addition to another group treated with sodium diclofenac (25 mg/kg) as a standard anti‐inflammatory and analgesic drug. Hind paw volume was determined at zero time (V_i_) and after 3 h (V_f_) using Plethysmometer, LE 7500 (Panlab, HARVARD Apparatus, Spain). Increase in paw volume was calculated as follows:

%Increaseofedema=[(Vf−Vi)/Vi]×100

Additionally, nociceptive threshold was quantified also with an analgesiometer (Ugo Basile, Comerio, Varese, Italy). The force, in grams, applied to the paw increased proportionally until the rat withdraws its paw.

#### 2.4.4 In Vivo Protection of MLE Against γ‐radiation

##### 2.4.4.1 Experimental Design

A total of 24 male Wistar rats were divided into 4 groups (*n* = 6) as follows; **control** group received vehicle (5% tween 80 suspended in distilled water) for 5 days, **Rad** group received vehicle (5 days) and exposed to whole body acute 6 Gy radiation dose at the third day, which was selected based on previous radiobiological studies demonstrating that this dose reliably induces acute oxidative stress, inflammatory responses, and histopathological alterations in radiosensitive organs [31, 32]. The third [**Rad + 300 MLE**] and fourth [**Rad + 500 MLE**] groups were irradiated as in second group and received MLE (300 & 500 mg /kg/ p.o./ day) for 5 days, respectively. Each animal's body weight (g) was recorded on the first and final days of the experiment. At the end of experiment (sixth day); all animals were anesthetized, then sacrificed by cervical dislocation. Whole blood, serum, and intestine tissue samples were used for biochemical investigations. Small parts of intestine, spleen, liver, and right kidney were dissected and stored in 10% formaldehyde for histopathological examinations.

##### 2.4.4.2 Gamma Irradiation

Irradiation was carried out at NCRRT, EAEA; using Gamma cell‐40 biological irradiator, Caesium‐137 source manufactured by the Atomic Energy of Canada, Limited at a dose rate of 0.33 Gy/min. The irradiator was calibrated annually by the Radiation Dosimetry Department, EAEA, with a reported dosimetry uncertainty of ±5%.

Animals were placed in well‐ventilated irradiation chambers and positioned at a fixed distance of 70 cm, ensuring uniform whole‐body dose distribution. Fixed structural lead shielding of the irradiation facility was used to minimize scattered radiation and ensure operator safety. All procedures were carried out in full compliance with Egyptian Atomic Energy Authority radiation protection regulations and approved institutional ethical guidelines.

##### 2.4.4.3 Histopathological Examinations

Liver, kidney, intestine, and spleen tissues specimens were fixed in 10% formalin then dehydrated with ethanol solution and cleared with xylene then embedded in paraffin. Serial 5 µm sections of the tissue were obtained and stained with hematoxylin‐eosin (HE). The histopathological sections were examined under a light microscope (Olympus CX31, Ireland) at 200x magnification by a pathologist who was blind to the study. The sections and the histological severity of the liver damage was graded using a Suzuki score. It comprised three components (sinusoidal congestion, hepatocyte vacuolation and necrosis), which were graded from 0 to 4. Histopathologic findings of intestinal sections were graded according to the Chiu scoring system where grade 0 represents Normal mucosal villi; grade I represents development of a subepithelial space, usually at the tip of the villus, with capillary congestion; grade II represents extension of the subepithelial space with moderate lifting of the epithelial layer; grade III represents massive epithelial lifting down the sides of villi; grade IV represents denuded villi with lamina propria, dilated capillaries exposed, increased cellularity of the lamina propria; and grade V represents digestion and disintegration of the lamina propria; hemorrhage and ulceration.

##### 2.4.4.4 Haematological Screening

Complete blood picture was assesed [Haemoglobin (Hg), hematocrit (Hcr), platlets count (PLTs), red blood cells (RBCs), and white blood cells (WBCs) counts] using automatic hematology analyzer HA‐VET (CLINDIAG systems, Pollare, Belgium).

##### 2.4.4.5 Assesment of Anti‐Inflammatory Activity (CRP & TNF‐α)

CPP and Tumor necrosis factor‐α (TNF‐α) was measured by using DuoSet ELISA kit [catalog; DY1744] and Biosource International ELISA Kit [catalog; ERA56RB], respectively, according to their manufacturer instructions.

##### 2.4.4.6 Immunohistochemical Staining of NF‐κB

Paraffin‐embedded intestinal tissue sections were deparaffinized, rehydrated, and subjected to heat‐induced antigen retrieval in 0.01 M citrate buffer (pH 6.0). Endogenous peroxidase activity was quenched with 3% hydrogen peroxide, followed by blocking of non‐specific binding. The sections were incubated overnight at 4°C with rabbit polyclonal anti‐NF‐κB p65/RelA antibody [catalogue; A2547, ABclonal Technology Co., USA] at a dilution of 1:300 according to the manufacturer's instructions. NF‐κB expression was quantified by measuring the optical density (OD) of the brown immunostaining in five randomly selected, non‐overlapping high‐power fields per intestinal section using ImageJ software (version 1.46a; National Institutes of Health, USA).

##### 2.4.4.7 Assessment of Antioxidant Activity (TAC, GSH, & TBARs)

Total antioxidant capacity (TAC) was determined based on the ability of antioxidants in the sample to inhibit oxidation of hydrogen peroxide, producing a coloured complex proportional to the total antioxidant capacity. Absorbance was measured at 505 nm according to the manufacturer's instructions [Bio‐Diagnostic, Giza, Egypt, catalogue; TA2513].

Glutathione (GSH) was determined based on the reaction of sulfhydryl groups in GSH with 5,5′‐dithiobis‐(2‐nitrobenzoic acid) (Ellman's reagent) to produce the yellow‐coloured product that was measured spectrophotometrically at 405 nm, according to the manufacturer's instructions [Bio‐Diagnostic, Giza, Egypt, catalogue; GR2511].

Thiobarbituric reactive substances (TBARs) was determined based on the reaction of malondialdehyde (MDA), a major lipid peroxidation product, with thiobarbituric acid (TBA) under acidic conditions and high temperature to form a pink chromogen, which was measured spectrophotometrically at 535 nm, according to the manufacturer's instructions [Bio‐Diagnostic, Giza, Egypt, catalog; MD2529].

##### 2.4.4.8 Assesment of Liver and Kidney Functions

ALT (alanine amino transfease) [catalog; AL1031], AST (aspartate amino transferase) [catalog; AS1061] and Cr (creatinine) [catalog; CR1250] were assesed in serum using colorimetric assay kits according to the manufacturer instructions [Biodiagnostic Co., Egypt].

### 2.5 Statistical Analysis

Sample size for each experiment was determined by using G Power version 3.1.9.4 software. The Shapiro–Wilk test was used to assess the entire data set for normality and homogeneity of variance. Data were expressed as mean ± standard error of mean (SEM). GraphPad Prism software program was used for carrying out all statistical tests using one‐way analysis of variance (ANOVA) test followed by Dunnett's or Tukey's post‐hoc test.

## 3 Results

### 3.1 MLE Acute Toxicity

An acute toxicity test was carried out by dosing mice with different MLE doses (100, 200, 500, 800, 1000, and 2000 mg/kg) to ensure extract safety. Toxicological screening showed a high safety margin of MLE up to 2000 mg/kg without any behavioural or weight changes or any recorded mortalities up to 14 days.

### 3.2 Antiinflammatory and Analgesic Efficacy of MLE in Paw Edema Rats

Measuring of paw edema volume and nociception pain in rats after carrageenan injection is considered a promising and suitable model for screening of anti‐inflammatory and analgesic activities of new drugs. The current preliminary screening of anti‐inflammatory and antinociceptive effects of MLE via carrageenan‐induced paw edema model showed a significant inhibition of paw edema by 28.8%, 45.9%, and 64.8%, at doses of 300, 500 and 1000 mg/kg, respectively (Table 1), with a noteicable significant reduction in paw edema by 64.3% upon treatment with sodium diclofenac. Interstingly, MLE exhibited antinociceptive effect at doses of 300, 500 and 1000 mg/kg compared to hyperalgesic group that evidenced by a significant increment in nociceptive threshold by 46.7%, 54.7% and 61.9%, respectively, where diclofenac showed a significant increase in nociception threshold by 63.3% (Table 1).

**TABLE 1:** Percentage increase in paw edema and nociceptive threshold (g) of Na diclofenac (25 mg/kg, p.o) or MLE at doses of 150, 300, 500, or 1000 mg/kg, p.o, in carrageenan induced paw edema and hyperalgesia in male rats (*n* = 6).

	paw edema increase %	Nociceptive threshold (g)
Normal	—	121.30 ± 0.76
Paw edema (PE)	74.76% ± 3.71	56.25 ± 1.31
PE + MLE (150 mg/kg)	64.22% ± 1.45	60.20 ± 1.97*
PE + MLE (300 mg/kg)	53.21% ± 3.51*	82.55 ± 0.85*
PE + MLE (500 mg/kg)	40.74% ± 2.56*	87.05 ± 0.42*
PE + MLE (1000 mg/kg)	26.32% ± 1.09*	91.08 ± 0.67*
PE+ Na diclofenac(25 mg/kg)	26.68% ± 0.84*	91.83 ± 8.59*

*Note*: Values are expressed as mean± SE. ANOVA was carried followed by Dunnette's post‐hoc test.

* Referred to significance from PE control group at *p *< 0.05.

Therefore, the minimum significant effective two doses of MLE (300 & 500 mg/kg) in carrageenan paw edema test, had been selected for further estimating the radioprotective efficacy of MLE.

### 3.3 Radioprotective Efficacy of MLE

#### 3.3.1 MLE Protection Against Weight Loss

In the current study, body weight was recorded at the baseline and at the end of experiment to assess the impact of whole‐body γ‐irradiation on body weight and to evaluate the dose‐dependent ability of MLE to preserve body weight as an indicator of its radioprotective activity. Exposure of rats to an acute γ‐radiation dose (6 Gy), induced a significant decrease in body weight by 7.5%, where the normal increment in body weight of normal control rats was 8.9%. It has been noticed that treatment with MLE at dose of 300 mg/kg decreased the rate of body weight loss to be 2.6%, interestingly, the higher dose of MLE (500 mg/kg) protected irradiated rats from losing weight and maintained normal weight gain to be 8.6% (Figure 2).

#### 3.3.2 MLE Protective Effect on Blood Component and Spleen

It is well‐known that the hematopoietic cells are very sensitive to γ‐radiation, which alter their number and function. Irradiated rats in the current study showed a significant suppression in WBCs count (47.7%) and platelets count (33.7%) (Table 2), as well as a massive reduction in white pulp follicles size of splenic tissue with severe depletion of lymphocytes (Figure 3A). Fortunately, minor improvements in lymphocytes depletion and WBCs count (14.3%) have been reached after treatment with 500 mg/kg MLE for 5 successive days (Table 2). In addition, the splenic tissue histopathological investigation of Rad + 500 MLE group showed less tissue congestion (Figure 3D).

**TABLE 2:** Effect of MLE  (300 and 500 mg/kg, p.o.) on haematological functions (haemoglobin, Hb; haematocrit, HCR; platelet count; WBC, white blood cell count; and RBC, red blood cell count) in 6 Gy irradiated rats (*n* = 6). *p* < 0.05.

	Hb (g/dl)	HCR (%)	Platlets count (x1000/cmm)	Total leuokocytic count (TLC) (x1000/cmm)	RBCs (x1000/µl)
**Control**	10.2 ± 0.9	27.7 ± 2.4	151 ± 10.3	6.7 ± 0.2	5.4 ± 0.5
**Rad**	12.2 ± 0.9	38.6 ± 2.8	100 ± 9.4*	3.5 ± 0.3*	6.4 ± 0.3
**Rad+** **300MLE**	11.2 ± 0.2	35.8 ± 1.4	109 ± 4.75*	3.8 ± 0.02*	6.2 ± 0.2
**Rad+** **500 MLE**	11.6 ± 0.3	36.4 ± 1.0	128 ± 3.63*, ^#^	4.0 ±0.03*	6.1 ± 0.1

*Note*: Values are expressed as mean ± SE. Data were analyzed by one‐way ANOVA followed by Tukey as a post‐hoc test.

* Significant from the control group at *p* < 0.05;

^#^ Significant from Rad group at *p* < 0.05.

#### 3.3.3 Systemic Anti‐inflammatory and Antioxidant of MLE

In the current work, MLE showed potential systemic antioxidant and anti‐inflammatory activities explored by improvement in serum TAC and blood GSH levels, as well as suppression of TBARs, CRP and TNF‐α serum levels (Figure 4). The Rad group showed significant decrements in serum TAC, and blood GSH contents by 23.9% and 36.9%, respectively, in addition to a significant increase in serum TBARs contents by 66.7%, compared to control normal values (Figure 4A–C). Interestingly, both doses of MLE (300 & 500 mg/kg) significantly increased serum TAC by 49.4% and 50.4%, respectively, in addition to increase in serum GSH content by 63.7% and 92.6%, respectively (Figure 4A,B). However, only the dose of MLE at 500 mg/kg showed a significant decrease in serum TBARs by 22.34% (Figure 4C). The serum CRP and TNF‐α levels were also investigated in the current study. The Rad group showed a significant increase in both CRP and TNF‐α levels by 54.6% and 779.3%, respectively, while treatment of irradiated rats with MLE at doses of 300 and 500 mg/kg exhibited potential anti‐inflammatory activity via significant suppression of CRP by 25.4% and 29.7%, respectively (Figure 4D). Furthermore, serum TNF‐α level significantly decreased in the Rad + 300 MLE and Rad + 500 MLE groups by 74.1% and 74.9%, respectively compared to Rad group (Figure 4E).

#### 3.3.4 MLE Protects Intestine Against Acute Radiation Effect

The gastrointestinal tract is one of the most radiation‐sensitive organs, as ROS triggered inflammatory mediators that proceeding to cells death. In the current study, acute irradiation of rats at 6 Gy showed a significant decrease in intestinal GSH by 18.8%, and significant increments in intestinal TBARs level by 65.9% (Figure 6A,B). However, this increment in intestinal oxidative stress status was followed by a subsequent significant increase in intestinal NF‐κB expression (Figure 5) and TNF‐α levels (Figure 6C) compared to non‐irradiated group by 484.2% and 326.9%, respectively. These mechanistic changes were accompanied with a partial necrosis at the tips of some villi and an infiltration of inflammatory cells, mainly lymphocytes and macrophages, into the lamina propria (Figure 7B). There was also observed shortening and atrophy of some villi, loss of goblet cells, necrosis of intestinal glands and notable sub‐epithelial edema, leading collectively to significant increase in intestinal damage scoring (Figure 7E). Interestingly, significant increments in intestinal GSH contents by 29.4% and 30.3%, had been recorded after treatment with MLE at 300 and 500 mg/kg doses, respectively compared to Rad group (Figure 6A). Alongside, a significant decrement of intestinal TBARs by 22.3% was recorded after treatment of irradiated rats with MLE at a dose of 500 mg/kg only (Figure 6B). Remarkably, treatment with MLE at both doses (300 & 500 mg/kg) showed a potential decrement in intestinal TNF‐α by 78.8% and 79.5%, respectively, in addition to a significant decrease in NF‐κB expression by 72.1% and 77.5%, respectively (Figure 5C,D and 6C). The protection power of MLE on GIT was sightsaw with histopathological investigation, especially at dose of 500 mg/kg, which exhibited significant decrease in intestinal damage scoring (Figure 7E), as evidenced by intact villus epithelial linings and minimal loss of goblet cells with a limited infiltration of inflammatory cells into the lamina propria (Figure 7D).

#### 3.3.5 MLE Improved Liver and Kidney Function

The current study showed also a significant increase in serum liver enzymes (ALT & AST) and serum Cr level in irradiated rats by 28.1%, 49.1%, and 22.7%, respectively compared to control normal rats (Table 3). Administration of MLE before and after radiation exposure showed significant decrements in ALT and AST activities by 10.1% and 41.4%, respectively at the dose of 300 mg/kg, and by 22.7% and 44.2%, respectively at the dose of 500 mg/kg (Table 3). Histopathological examination supported these findings that explored revealed vacuolation, nuclear pyknosis of hepatocytes, and extensive necrosis of the hepatic lobules in Rad group. Additionally, disorganization of hepatic cords, hyperplasia of Kupffer cells, and congestion of the central vein along with hepatic sinusoids [grade 4], Figure 8IB. Administration of MLE at dose of 300 mg/kg showed ballooning of hepatocytes and narrowing of the hepatic sinusoids and numerous pyknotic nuclei of hepatocytes, along with a few apoptotic bodies in the centrilobular zone [grade 2], Figure 8IC. Interestingly, treatment of irradiated rats with 500 mg/kg MLE exhibited regeneration of hepatic cells that revealed by a limited number of nuclear pyknosis and narrowing of the hepatic sinusoids [grade 1], Figure 8ID.

**TABLE 3:** Effect of MLE (300 and 500 mg/kg, p.o.) on serum ALT (IU/L) and AST (IU/L) activities and Cr level (mg/dl) in 6 Gy irradiated rats (*n* = 6).

	ALT (IU/L)	AST (IU/L)	Cr (mg/dl)
Control	25.62 ± 0.65	22.77± 0.86	1.031 ± 0.06
Rad	32.83 ± 1.42*	33.85 ± 1.71*	1.26 ± 0.02*
Rad + 300 MLE	29.51 ± 1.82#	19.83 ± 0.59#	1.26 ± 0.02
Rad+ 500 MLE	25.37 ± 1.12#	18.89 ± 0.47#	1.083 ± 0.07

Values are expressed as mean ± SE. Data were analyzed by one‐way ANOVA followed by Tukey as a post‐hoc test.

* Significant from the control group at *p* < 0.05;

^#^ significant from the Rad group at *p* < 0.05.

**FIGURE 8I:**
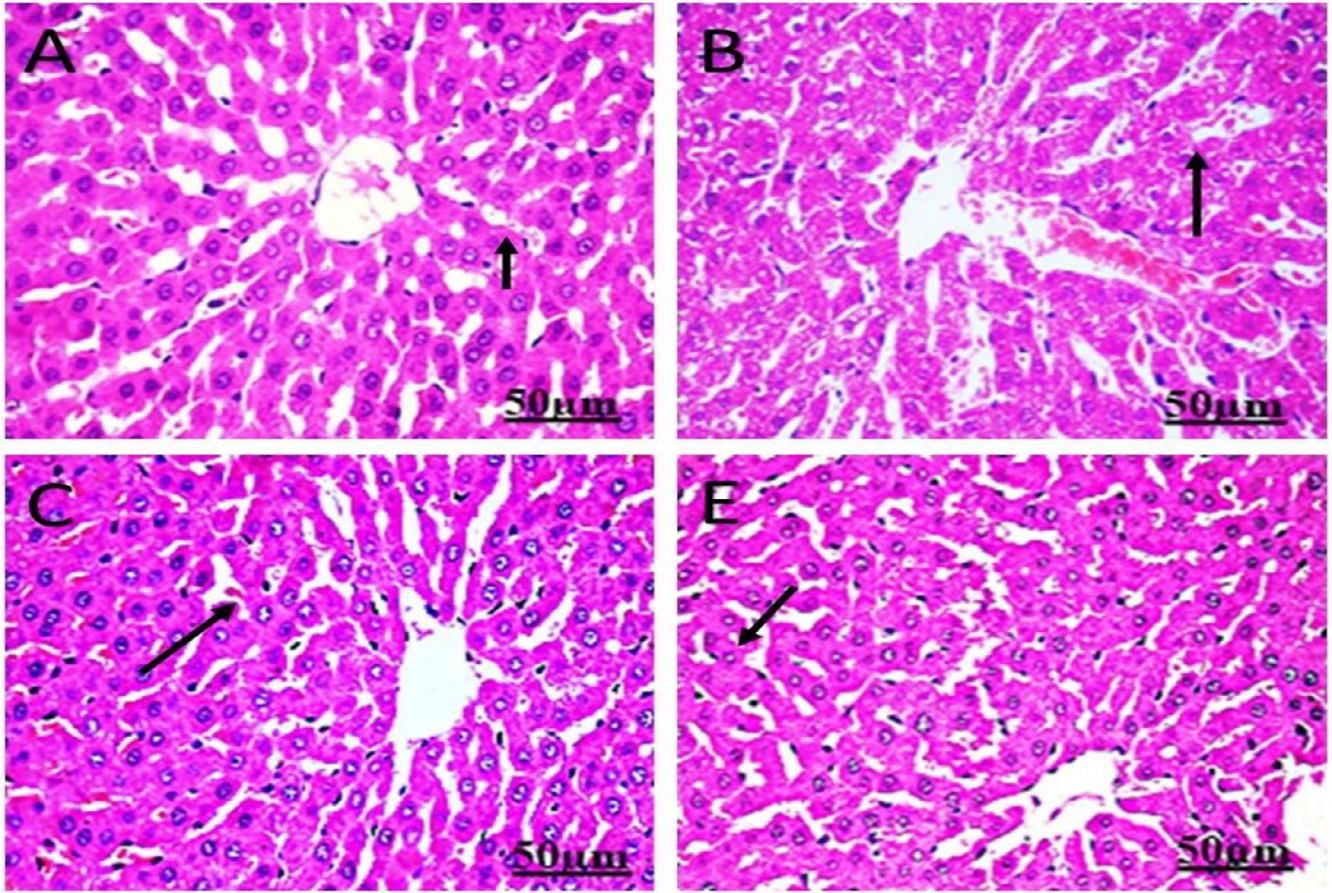
Photomicrograph of hepatic tissue section showing (A) normal architecture of hepatic lobule in the control group, (B) centrilobular coagulative necrosis of hepatocytes in the Rad group, (C) ballooning of hepatocytes and narrowing of hepatic sinusoids in the Rad ± 300 MLE group, and (D) regeneration of hepatic cells which appeared deeply basophilic and numerous numbers of binucleated cells in the Rad ± 500 MLE group (H&E X200).

In addition, MLE exerted a measurable protective effect against radiation‐induced renal injury, particularly at the higher dose (500 mg/kg). This was evidenced by a reduction in serum creatinine levels by 4.75% compared with the irradiated group (Table 3), indicating partial preservation of renal function. Histopathological examination further supported this finding (Figure 8II), where irradiated animals exhibited marked tubular epithelial necrosis and intraluminal eosinophilic casts. In contrast, MLE treatment, in particularly at 500 mg/kg, attenuated these pathological alterations, as reflected by improved renal architecture with only mild tubular epithelial cell swelling.

**FIGURE 8II:**
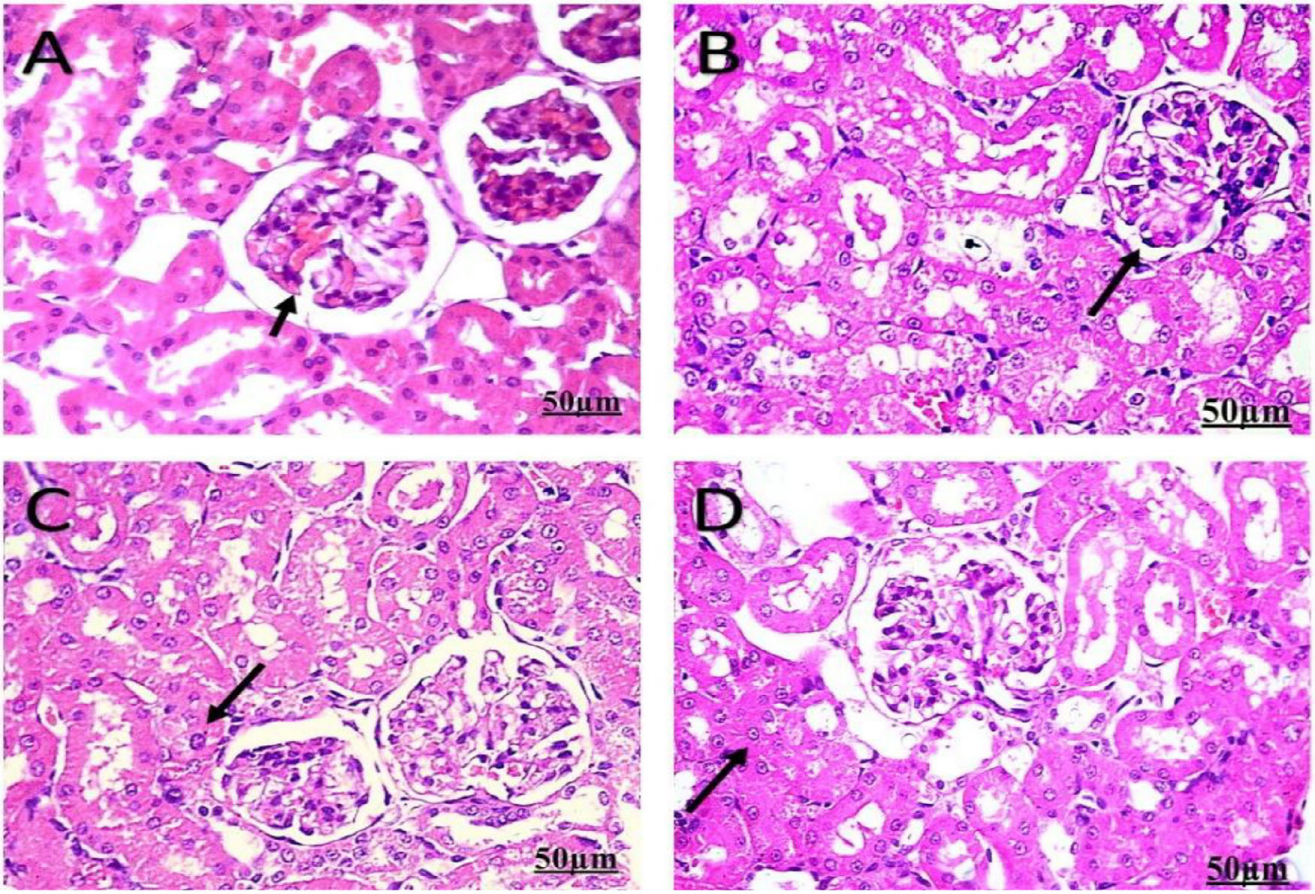
Photomicrograph of kidney tissue section showing (A) normal histological structure of glomeruli and renal tubules in control group, (B) tubular epithelial necrosis and desquamation in Rad group, (C) focal granulovacuolar epithelial cell degeneration in Rad ± 300 MLE group, and (D) mild swelling of tubular epithelial cells in Rad ± 500 MLE group (H&E X200).

## 4 Discussion

The relevance of depending on natural extracts to avoid the harmful and long‐term negative consequences of radiation exposure has come to light in recent years. Conventional thiol synthetic radioprotectors have seen limited application due to their undesirable effects, instead current investigations into natural sources have demonstrated similar radioprotective efficacy with diminished negative consequences [33]. In the current study, *Malphigia glabra L*. leaves had been targeted to evaluate their potential radioprotective effect, after their whole aqueous extraction. Toxicological screening of MLE showed high safety margin up to 2000 mg/kg, which was in agreement with another study that recorded the safety of ethanolic extract of *Malphigia glabra L*. leaves up to 5 g/kg [17].

Controlling and managing of inflammatory conditions brought on by radiation exposure attracted numerous studies over the years to find a natural anti‐inflammatory herbal extract to sidestep severe medical conditions and cells death triggered by inflammatory status [1]. The significant anti‐inflammatory and antinociceptive activities of MLE in inflamed and hyperalgesia rats’ paw could be attributed to the current phytochemical findings of the constituents of MLE including kampferol, afzelechin, and engeletin derivatives that had been reported before for their individual and potential anti‐inflammtory activities [34, 35, 36, 37]. Additional explanation of the antiinflammatory activity of MLE is based on its powerful savengening of free radicals activity [16], as explored by studies carried on antioxidant medicinal plants that returned the anti‐inflammatory activities of these plants because of their scavengening of free radiacals and blocking of ROS accumulation which is essential for activation of NF‐kB, the regulator factor of inflammatory cascade [3]. On the other side, antioxidants has a role in pain modulation throught inhibition of oxidative stress, which is accountable for metabolic changes that cause peripheral and cerebral nociceptive stimulation [38].

After exhibition of significant anti‐inflammatory activity, MLE was further evaluated for its radioprotective activity on acute irradiated rats (6 Gy). Changes in body weight were screened before and after the experiment, as exposure to γ‐radiation was usually accompanied by weight loss, a problem which has to be controlled to ensure the efficacy of the treatment plan [39]. The same finding has been established in the current work, where the acute exposure to γ‐radiation induced a significant decrease in body weight. Notably, high‐dose MLE treatment markedly attenuated body‐weight loss and preserved near‐normal weight gain. While antioxidant‐rich plant extracts have been reported to ameliorate cancer treatment‐induced cachexia [40], alternative, non‐mutually exclusive explanations should also be considered, including improved food intake, reduced stress burden, and overall health stabilization, in addition to potential direct pharmacological effects.

It is well‐known that the haematopoietic cells are very sensitive to γ‐radiation, which alters their number and function [41]. In the current study, the Rad group showed a significant lowering of total leukocyte count, platelet count and HCR. However, investigation of the blood picture after treatment with a higher dose of MLE revealed only a mild preservation of the WBC count and improvement in the histopathological examination of spleen tissue. The delay in improvement of other haematological parameters could be explained by and returned to the short treatment duration that wasn't enough to regain the normal count of WBCs. This was agreed with the study of Sanguri and Gupta [42], which discussed that it could take at least 30 days for blood cells to reach the normal blood count. Nevertheless, the observed protection against spleen changes and weight loss in the Rad+ 500 MLE group might be attributed to the richness of MLE with phenolic and flavonoid contents, which had a great impact on enhancing immunity and improving general health during radiation exposure [18].

The antioxidant and anti‐inflammatory activities of MLE were explored in the current study after enhancement in TAC and GSH levels, as well as suppression of TBARs, CRP, and TNF‐α levels in irradiated rats. These results accomodate with our previous work that exhibited a strong scavengering activity of MLE following DPPH screening because of its high phenolic and flavonoids content [17]. Furthermore, the current isolated compounds from MLE extract were derived from strong free radical scavengering comounds such as triterpenoid (isoarborinol), catechins (Afzelechin), flavanonol rhamnoside (Engeletin), and flavonoids (Kampferol), which elucidated the commanding MLE antioxidant activity [43, 44, 45]. Kaempferol had a poweful antioxidant activity as evidenced by enhancement of cellular glutathione reductase and peroxidase [46]. Additionally, afzelechin has the efficacy in reducing MDA level and increasing antioxidant enzymes such as glutathione trductase and superoxide dismutase [47].

Moreover, MLE significantly supressed serum CRP and TNF‐α levels in irradiated rats. However, suppression of CRP is a promising target for patients received radiation therapy as the elevation of CRP is strongly correlated to the patients' degrees of pain, which may have an impact on patients' survival [48]. The anti‐inflammatory efficacy of MLE could be mainly returned to its content of kaempferol 7‐O‐β‐D‐glucopyranose, kaempferol 3‐O‐Rutinoside, afzelechin, engeletin and other flavonoid compunds, which characterized by their significant anti‐inflammtory activity via the suppression of IL‐18, IL‐1β, IL‐6 and TNF‐α and inhibition of NF‐kB, Toll like receptors‐4 [TLR4] and COX‐2 enzyme [34, 35, 36, 37].

The gastrointestinal tract is the earliest and noticeably affected organs following radiation exposure as the adverse effects could be extended for years. In the current study, intestinal injury following acute radiation exposure was accompanied by a noticeable oxidative stress status alongside with elevation of TNF‐α level and NF‐kB expression. The early acute injury of intestine by radiation exposure was mainly due to mucosal injury by ROS that was further aggregated cytokines and induced gut dysbiosis, leading finally to the gut cells' apoptosis [5, 6]. Interestingly, treatment with MLE, especially at 500 mg/kg, elevated the GSH intestinal content, and significantly suppressed TBARs, TNF‐α and NF‐kB expression with a remarkable improvement in intestinal villi. The established anti‐inflammatory properties of most flavonoids was in consistent with our findings as flavonoids are recognized as multifunctional phytochemicals capable of attenuating inflammatory responses by suppressing the activation of NF‐κB, inhibiting the production of pro‐inflammatory cytokines, including TNF‐α, IL‐1β, and IL‐6, and reducing oxidative stress through the scavenging of reactive oxygen species. These mechanisms are particularly relevant in radiation‐induced tissue injury, where excessive ROS generation and sustained inflammatory signaling contribute to cellular damage. Therefore, the observed reduction in TNF‐α and NF‐κB expression following MLE treatment is likely returned to its high flavonoid content, supporting previous reports that flavonoid‐rich plant extracts provide protection against inflammation‐associated tissue injury [49]. However, the gastroprotective impact of MLE could be discussed by the existence of engeletin which showed a high antioxidant efficacy that attenuated GIT inflammation by targeting AMPK/ SIRT signaling pathway, thus maintenance the mitochondrial function [50]. In addition, kaempferol which specified by phenyl rings and four hydroxyl substituents structure, has an excellent free radical scavenging activity that mitigated inflammatory reaction, making it a potential gut protector [51]. The afzelechin content in MLE could participated in that anti‐inflammatory activity, where afzelechin showed a potential modulation of pathological inflammation via suppression of COX‐2/PGE2 pathway with a consequent decrement of TNF‐α level [36]. Additionally, β‐isoarborinol, a pentacyclic triterpenoid identified in the current extract, has been reported to exhibit anti‐inflammatory potential. Recent metabolomic and in silico investigations have suggested that β‐isoarborinol may interact with inflammatory mediators involved in the NF‐κB signaling cascade, supporting its potential role in modulating inflammatory responses. Therefore, the observed biological activity of MLE could be as a result of the synergistic actions of flavonoids together with triterpenoid constituents, rather than being attributable to a single class of phytochemicals [52].

It is well agreed that adverse effects of γ‐radiation were not only minored to hematopoietic and gastrointestinal systems, but it could be extended to induce hepatic and renal tissue injuries [7]. Our results showed a significant elevation in serum ALT, AST and serum Cr levels in Rad group compared to control normal group. Additionally, necrotic hepatocytes and marked tubular epithelial necrosis and intraluminal eosinophilic casts had been remarked in the hepatic and renal histopathological examination. Accumulation of ROS post radiation triggered inflammatory reaction that further proceeded to cell apoptosis and necrosis leading finally to organs dysfunction [3, 7]. Treatment of irradiated rats with MLE maintained normal liver and kidney functions, especially at the higher MLE dose. This was in line with prior research that demonstrated the hepatoprotective effectiveness of ethanolic extract of *Malphigia glabra L*. leaves due to their potential anti‐inflammatory and antioxidant properties [10]. Furthermore, the flavonoid constituents of MLE including kaempferol, engeletin and afzelechin have been reported for their hepatoprotective effects against different hepatotoxic agents via variable antioxidant and anti‐inflammatory signaling pathways [47, 53, 54]. In addition, kaempferol has the ability of activating Nrf‐2/Ho‐1/antioxidants axis in kidney tissue, which in turn led to suppression of NF‐κB, TNF‐α and IL‐6, thus protecting nephrotic cell from final apoptosis [55]. However, although little changes in serum creatinine had been observed after MLE treatment, significant improvements in renal function were emphasized by improvement of kidney histopathological examination. Furthermore, creatinine serum is considered relatively insensitive marker of early or mild renal injury, and small changes within the normal range may not reflect biologically significant improvements in renal function [56].

Despite the protective effects demonstrated in the present study, several limitations should be acknowledged. Additional mechanistic analyses of oxidative stress‐ and inflammation‐related signaling pathways, will be incorporated in the future study, in particularly, the activities of endogenous antioxidant enzymes, such as superoxide dismutase (SOD) and catalase (CAT), to elucidate a more robust mechanistic interpretation of MLE‐mediated radioprotection.

## 5 Conclusion

The present study demonstrated that *Malpighia glabra* L. leaves extract (MLE), rich in kaempferol, engeletin, afzelechin, ascorbic acid, and other flavonoids in addition to β‐isoarborinol exerted significant antioxidant and anti‐inflammatory activities and provided significant protection against acute γ‐radiation‐induced systemic inflammation and tissue injury in experimental rats. MLE attenuated oxidative stress, suppressed inflammatory mediators, reduced NF‐κB expression, and preserved the histological architecture and function of multiple abdominal organs, including the intestine, liver, kidney, and spleen. These findings support the potential of MLE as a safe natural radioprotective agent.

Nevertheless, the study is limited by the absence of comprehensive molecular analyses of oxidative stress‐ and inflammation‐related signaling pathways. Future studies should incorporate these mechanistic biomarkers, evaluate long‐term radioprotective efficacy, and investigate the individual contributions of the major bioactive constituents to further validate the therapeutic potential of MLE.

## Author Contributions

**A. M. Fekry**: plant extraction, chemical analysis, compounds isolation and methodology. **W. A. Elsabbagh**: methodology, biological evaluation, statistical analysis and supervision. **A. M. Fekry** and **W. A**. **Elsabbagh**: writing the original draft. **M. S. Abu Bakr, A. I. Mohammed,** and **M. A. El‐Ghazaly**: validation and supervision. All authors shared conceptualisation, resources, experimental designing, investigation, visualisation, reviewing and editing.

## Funding

The authors reported that there is no funding associated with the work included in this article.

## Conflicts of Interest

The authors declared no conflicts of interest.

## Supporting information




**Supporting File 1**: cbdv71636‐sup‐0001‐SuppMat.docx

## Data Availability

All data generated or analyzed during this study are included in this article and supporting file.
