# Supplementary material for: Extraction and Isolation of Bioactive Compounds From Malpighia glabra L. Leaves: In Vivo Investigation of the Leaf Extract Against Oxidative Stress and Inflammation Triggered by γ‐Radiation
Source: Chem Biodivers. 2026 Sep 8;23(9):e71636. doi: 10.1002/cbdv.71636 (PMC13551133; doi:10.1002/cbdv.71636)
Supplement: Supplementary file 1 [file CBDV-23-e71636-s001.docx]

**Supplementary file**

**Table S1:^1^H and ^13^C NMR spectral data of compound 1(400 MHz for ^1^H and 100 MHz for ^13^C, Pyridine-*d_5_*):**

| **compound 1** | | | |
| --- | --- | --- | --- |
| **Position** | **^1^H (J in Hz)** | **^13^C** | **HMBC** |
| 1 |  | 36.863 |  |
| 2 |  | 28.723 |  |
| 3 | 3.50, dd, 9.5, 3.8 | 78.346 | C-23, 24 |
| 4 | - | 39.961 |  |
| 5 |  | 53.104 |  |
| 6 |  | 22.069 |  |
| 7 |  | 27.315 |  |
| 8 | 1.50, m | 41.425 | C-9, 11 |
| 9 | - | 149.523 |  |
| 10 | - | 40.283 |  |
| 11 | 5.11, brs | 114.570 | C-8 |
| 12 |  | 36.863 |  |
| 13 | - | 37.218 |  |
| 14 | - | 38.653 |  |
| 15 |  | 30.035 |  |
| 16 |  | 36.484 |  |
| 17 | - | 43.270 |  |
| 18 |  | 52.508 |  |
| 19 |  | 20.632 |  |
| 20 |  | 28.923 |  |
| 21 |  | 59.943 |  |
| 22 |  | 31.204 |  |
| 23 | 1.28, s | 29.169 | C-3, 4, 5, 24 |
| 24 | 1.13, s | 16.814 | C-3, 4, 5, 23 |
| 25 | 1.15, s | 22.526 | C-1, 5, 9, 10 |
| 26 | 0.89, s | 17.502 | C-8, 13, 14, 15 |
| 27 | 0.83, s | 15.787 | C-12, 13, 14, 18 |
| 28 | 0.78 (3H, s, H-28) | 14.401 | C-16, 17, 18, 21 |
| 29 | 0.88 | 23.416 | C-21, 22, 30 |
| 30 | 0.94, d, 5.6 | 22.694 | C-21, 22, 29 |

**Table S2: ^1^H and ^13^C NMR spectral data of compounds 2,3,4,5 and 6 (400 MHz for ^1^H and 100 MHz for ^13^C, DMSO-*d_6_*):**

|  | **compound 2** | | **compound 3** | | **compound 4** | | **compound 5** | | **compound 6** | |
| --- | --- | --- | --- | --- | --- | --- | --- | --- | --- | --- |
| **^p^** | **^1^H** | **^13^C** | **^1^H** | **^13^C** | **^1^H** | **^13^C** | **^1^H** | **^13^C** | **^1^H** | **^13^C** |
| 2 | 4.52, d ,8 | 80.98 | 5.28, d, 10.4 | 81.57 | 4.71, d, 6.8 | 79.44 | - | 147.18 | - | 156.47 |
| 3 | 3.88, m | 66.28 | 4.74, d, 10.4 | 76.03 | 3.85, brs | 73.58 | - | 136.15 | - | 133.21 |
| 4α | 2.70, dd, 16.0, 5.2 | 28.27 | - | 194.70 | 2.78, d,11.6 | 27.23 | - | 176.28 | - | 177.34 |
| 4β | 2.35 , dd, 16.0, 8.4 |  |  |  | 2.56, m |  |  |  |  |  |
| 5 | - | 156.14 | - | 163.50 | - | 156.76 | - | 160.55 | - | 161.19 |
| 6 | 5.68, brs | 95.16 | 5.90, brs | 96.23 | 5.80, brs | 96.02 | 6.42, s | 99.03 | 6.19 , brs | 98.83 |
| 7 | - | 156.47 | - | 167.46 | - | 157.18 | - | 166.15 | - | 164.53 |
| 8 | 5.89, brs | 93.85 | 5.88, brs | 95.24 | 5.98, brs | 94.63 | 6.80, s | 92.03 | 6.40, brs | 93.80 |
| 9 | - | 155.35 | - | 162.19 | - | 155.78 | - | 155.89 | - | 156.8 |
| 10 | - | 99.13 | - | 100.93 | - | 100.84 | - | 105.05 | - | 103.91 |
| 1’ | - | 129.94 | - | 126.60 | - | 129.79 | - | 121.41 | - | 120.89 |
| 2’ | 7.14, d, 8 | 128.56 | 7.33, d, 8.4 | 129.13 | 7.20, d, 7.6 | 128.79 | 8.08, d, 8.4 | 129.74 | 7.98, d, 8.4 | 130.88 |
| 3’ | 6.73, d, 8 | 114.78 | 6.79, d, 8.4 | 115.25 | 6.79, d, 7.2 | 115.61 | 6.94 , d, 8.4 | 115.33 | 6.88, d, 8.0 | 115.10 |
| 4’ | - | 156.93 | - | 157.92 | - | 157.57 | - | 159.55 | - | 159.99 |
| 5’ | 6.73, d, 8 | 114.78 | 6.79, d, 8.4 | 115.25 | 6.79, d, 7.2 | 115.61 | 6.94, d , 8.4 | 115.33 | 6.88, d , 8.0 | 115.10 |
| 6’ | 7.14, d, 8 | 128.56 | 7.33, d, 8.4 | 129.13 | 7.20, d, 7.6 | 128.79 | 8.08, d, 8.4 | 129.74 | 7.98 , d, 8.4 | 130.88 |
| 1’’ | - | - | 4.15, d, 2.4 | 100.39 | 4.22, brs | 99.27 | 5.07, d, 6.8 | 100.02 | 5.3, d, 6.4 | 101.38 |
| 2’’ | - | - | 3.32, m | 70.14 | 3.33-3.54, m | 70.84 | 3.18-3.73 | 72.99 | 3.04-3.70 | 74.18 |
| 3’’ | - | - | 3.40, dd, 9.2, 2.8 | 70.47 | 3.33-3.54, m | 71.17 | 3.18-3.73 | 76.30 | 3.04-3.70 | 76.38 |
| 4’’ | - | - | 3.12, t, 9.4 | 71.70 | 3.33-3.54, m | 72.52 | 3.18-3.73 | 69.59 | 3.04-3.70 | 69.93 |
| 5’’ | - | - | 3.92, dq, 9.2, 6.4 | 69.07 | 3.33-3.54, m | 69.48 | 3.18-3.73 | 77.24 | 3.04-3.70 | 75.75 |
| 6’’a  6’’b | - | - | 1.05, d, 6.4 | 17.82 | 1.17, d, 6.4 | 18.36 | 3.18-3.73 | 60.59 | 4.38 | 66.89 |
| 1’’’ | - | - |  | - | - | - | - | - | 4.38, s | 100.78 |
| 2’’’ | - | - |  | - | - | - | - | - | 3.04-3.70 | 70.30 |
| 3’’’ | - | - | - | - | - | - | - | - | 3.04-3.70 | 70.61 |
| 4’’’ | - | - | - | - | - | - | - | - | 3.04-3.70 | 71.83 |
| 5’’’ | - | - | - | - | - | - | - | - | 3.04-3.70 | 68.26 |
| 6’’’ | - | - | - | - | - | - | - | - | 0.98, d, 6.0 | 17.74 |
| OH | 9.37, s | - | 9.88 | - | 9.47, brs | - | - | - | 9.01, brs | - |
| OH | 9.19, s | - | 11.84 | - | 9.36, brs | - | 10.19 , brs | - | 9.29, brs | - |
| OH | 8.94, s | - | - | - | 9.53, brs | - | 12.49, brs | - | 12.55, brs | - |
| OH | 4.88, d, 4.8 | - | - | - | - | - |  | - |  | -- |

**Figure 1S**. ^13^C (1a), ^1^H (1b) NMR and HMBC of compound 1, ß-Isoarborinol.

**Figure 2S**. ^13^C (2a) and ^1^H (2b) NMR of compound 2, Afzelechin.

**Figure 3S**. ^13^C (3a) and ^1^H (3b) NMR of compound 3, Engeletin.

**Figure 4S**. ^13^C (4a) and ^1^H (4b) NMR of compound 4, Afzelechin 3-O-Rhamnoside.

**Figure 5S**. ^13^C (5a) and ^1^H (5b) NMR of compound 5, Kaempferol 7-O-β-D- glucopyranose.

**Figure 6S**. ^13^C (6a) and ^1^H (6b) NMR of compound 6, Kaempferol 3-O-Rutinoside.

**a [ß-isoarborinol]**

**b [Afzelechin]**

**c [Engeletin]**

**d [Afzelechin 3-O-Rhamnoside]**

**e [Kaempferol 7-O-β-D- glucopyranose]**

**f [Kaempferol 3-O-Rutinoside]**

**Figure 7S**. MS/MS spectra of [a] ß-Isoarborinol, [b] Afzelechin, [c] Engeletin, [d] Afzelechin 3-O-Rhamnoside, [e] Kaempferol 7-O-β-D- glucopyranose, and [f] Kaempferol 3-O-Rutinoside.
